# Tellurium notebooks—An environment for reproducible dynamical modeling in systems biology

**DOI:** 10.1371/journal.pcbi.1006220

**Published:** 2018-06-15

**Authors:** J. Kyle Medley, Kiri Choi, Matthias König, Lucian Smith, Stanley Gu, Joseph Hellerstein, Stuart C. Sealfon, Herbert M. Sauro

**Affiliations:** 1 Department of Bioengineering, University of Washington, Seattle, Washington, United States of America; 2 Institute for Theoretical Biology, Humboldt University of Berlin, Berlin, Germany; 3 eScience Institute, University of Washington, Seattle, Washington, United States of America; 4 Department of Neurology and Center for Advanced Research on Diagnostic Assays Icahn School of Medicine at Mount Sinai, New York, New York, United States of America; UCSD, UNITED STATES

## Abstract

The considerable difficulty encountered in reproducing the results of published dynamical models limits validation, exploration and reuse of this increasingly large biomedical research resource. To address this problem, we have developed Tellurium Notebook, a software system for model authoring, simulation, and teaching that facilitates building reproducible dynamical models and reusing models by 1) providing a notebook environment which allows models, Python code, and narrative to be intermixed, 2) supporting the COMBINE archive format during model development for capturing model information in an exchangeable format and 3) enabling users to easily simulate and edit public COMBINE-compliant models from public repositories to facilitate studying model dynamics, variants and test cases. Tellurium Notebook, a Python–based Jupyter–like environment, is designed to seamlessly inter-operate with these community standards by automating conversion between COMBINE standards formulations and corresponding in–line, human–readable representations. Thus, Tellurium brings to systems biology the strategy used by other literate notebook systems such as Mathematica. These capabilities allow users to edit every aspect of the standards–compliant models and simulations, run the simulations in–line, and re–export to standard formats. We provide several use cases illustrating the advantages of our approach and how it allows development and reuse of models without requiring technical knowledge of standards. Adoption of Tellurium should accelerate model development, reproducibility and reuse.

## Author summary

There is considerable value to systems and synthetic biology in creating reproducible models. An essential element of reproducibility is the use of community standards, an often challenging undertaking for modelers. This article describes Tellurium Notebook, a tool for developing dynamical models that provides an intuitive approach to building and reusing models built with community standards. Tellurium automates embedding human–readable representations of COMBINE archives in literate coding notebooks, bringing to systems biology this strategy central to other literate notebook systems such as Mathematica. We show that the ability to easily edit this human–readable representation enables users to test models under a variety of conditions, thereby providing a way to create, reuse, and modify standard–encoded models and simulations, regardless of the user’s level of technical knowledge of said standards.

This is a *PLOS Computational Biology* Software paper.

## Introduction

Multiscale dynamical simulation requires the ability to build large, comprehensive, and complex models of biological systems. Examples include the *Mycoplasma genitalium* whole–cell model [1] and the central metabolism of *E. coli* [2]. These models are often composed of many submodels. Typically, submodels are developed and validated by other research teams. Indeed, without the ability to reuse existing models, constructing larger models becomes impractical.

Being able to reuse tools and techniques developed by others is a hallmark of science. Poor reproducibility of biomedical experimental studies has been recognized as a major impediment to scientific progress [3, 4]. Much of the focus on poor reproducibility has been on wet lab experiments. However, barriers to reproducibility is also a significant problem in computational studies [5–10]. In recognition of this problem, **reproducibility** has become a central focus of scientific software [11, 12]. The general experience of researchers in the field of modeling suggests that a similar problem in poor reproducibility also exists for biomodels. Difficulty in model reproducibility can result from a published model not being deposited in a public repository or from differences in the deposited model and the actual model used for published simulations. In addition, it is difficult for researchers to utilize and modify public models because the standards are not human–readable. This state of affairs imperils continued progress with developing and exploiting biological models.

We propose that reproducible computational studies must satisfy two requirements. First, they must be *transparent*; that is, researchers must be able to inspect and understand the details of the model and the computational experiments. With transparency, researchers can check assumptions and explore variations in computational studies. Second, computational studies must be *exchangeable*; that is, it must be possible for a study done in one computational environment to be done in another computational environment and produce comparable results. For a study to be exchangeable means that other researchers can make use of and build on the published results in their computational environment.

In order to be transparent and exchangeable, a computational model and any simulation experiments must be encoded in a standard format that separates the reusable part of a model and its simulations (i.e., parameters, processes, and kinetics) from the implementation used to simulate it (i.e., the numerical methods and algorithms used to generate results). Models can be described using the Systems Biology Markup Language (SBML) [13] or CellML [14] standards. These standards support models based on ordinary differential equations (ODEs), stochastic master equations, and constraint-based modeling [15, 16], partial differential equations (PDEs, using the proposed geometry extension [17, 18]), etc. Simulations can be described using the Simulation Experiment Description Markup Language (SED–ML) [19], which encodes the types of simulations, either time-course simulations or steady state computations, that should be run on a model. SED–ML allows specifying the exact numerical algorithms needed to run a simulation using the Kinetic Simulation Algorithm Ontology (KiSAO) [20], which includes widely used ODE (e.g., LSODA [21, 22], CVODE [23]) and stochastic solvers (e.g., Gillespie direct method [24], Gibson algorithm [25]).

In order to facilitate exchanging models and simulations between software tools, SED–ML simulations and SBML/CellML models can be packaged together using COMBINE archives [26]. However, few authoring tools exist for SED–ML and COMBINE archives [27, 28]. Furthermore, existing resources require technical knowledge of standards, restricting use of these standards by the modeling community at large. Therefore, an authoring tool is needed that allows a wider range of users to create and edit COMBINE archives containing both models and simulations. We propose that the authoring tool should satisfy five requirements:

1. It should represent the models or simulation specifications in a human–readable form.
2. It should allow the user to easily edit this human–readable representation.
3. It should allow the user to provide narrative, annotations, or comments in order to improve transparency.
4. It should translate the specifications into an implementation that can be used to run simulations.
5. It must be capable of repackaging the model and/or simulation in a standard form that is usable by other tools.

To address these requirements, we have developed the Tellurium Notebook environment, which extends the literate notebook concept used by tools like Jupyter [29] and Mathematica [30] to support community standards in systems biology. Whereas Jupyter notebooks contain code and narrative cells, Tellurium adds a third cell type for representing models and simulations encoded as standards. Our tool allows modeling studies to be constructed in a notebook environment and exported using community standards. This workflow provides both transparency, through a literate notebook, and exchangeability, through seamless, fluid support for standards.

Tellurium supports embedding human–readable representations of SBML [31] and SED–ML [32] directly in cells. These cells can be exported as COMBINE archives which other tools can read. We refer to this human–readable representation as *inline OMEX* (after Open Modeling and EXchange, the encoding standard used by COMBINE archives). Inline OMEX cells operate in much the same way as code cells, i.e., they have syntax highlighting and are executable. Executing an inline OMEX cell runs all SED–ML simulations in the cell, producing any plots or reports declared in the SED–ML. A major advantage of this approach is that it offers a means of authoring transparent, exchangeable modeling studies without requiring technical knowledge of file format standards.

## Design and implementation

Computational tools in systems biology typically focus on one of three major areas: authoring models, simulating models, or visualizing network–based depictions of models. Tellurium is primarily a tool for authoring and simulating models. As such, it must satisfy certain requirements in order to be useful for model construction. To construct a dynamical model, it is necessary to translate biological measurements and observations into a mathematical language that can be used to derive a set of differential equations. One method of constructing dynamical models in systems biology is to survey the literature for known interactions, rate constants, and parameters (the so–called “bottom-up” approach [33]). Thus, known information about a system can be used to construct a list of reactions, concentrations, and kinetic parameters, which, in turn, can be used to derive a set of differential equations. However, the list of known kinetic parameters is usually incomplete. Depending on the available data, some parameters may have to be inferred indirectly, while others may be completely unknown. A common method of addressing this problem is to perform parameter fitting, i.e., use a numerical optimization algorithm to minimize the discrepancy between the model’s predictions and available data by tuning parameters. To be useful, fitting parameters requires prudent selection of which parameters to optimize, as well as the numerical algorithm(s) to use and the upper and lower bounds for the selected parameters. As a result, parameter fitting can require considerable expertise and forethought, although attempts have been made to systemitize the process [34].

Tellurium’s principal feature is that it allows integrating standards such as SBML, CellML, and SED–ML in a single, unified representation called inline OMEX cells. This approach has the advantage of allowing users to encode models in these standards without necessarily being technical experts in said standards. While we could in principle have provided separate cell types for Antimony and PhraSEDML, we believe a single, unified editable representation is a better solution for three reasons: 1) With a unified representation, users are not required to know the technical boundaries between standards, 2) exchanging multiple related files between software tools is cumbersome and error–prone, hence a single cell should be exportable as a COMBINE archive, and 3) spreading out model and simulation specifications across different cells could lead to synchronization issues if a cell is updated but not re–executed. Therefore, we believe that having a single cell type which is interconvertible with a COMBINE archive provides the best way to author and exchange modeling studies.

In order to provide a unified representation of a COMBINE archive, it is necessary to integrate many standards and software technologies. Table 1 shows the roles of the various standards and technologies included in Tellurium. While the components in Table 1 can, in principle, be used independently from Tellurium, they do not carry the same benefits for reproducibility when used in isolation. Without integration, a user would need to be familiar with the application programming interface (API) of each of the libraries in the workflow above.

**Table 1:** Standards and terminology at a glance.

Standard / Technology	Description
SBML and CellML	Standards and respective file formats for systems biology models. Originally designed to support ODE models, but have since expanded to cover other types of models.
SED–ML	Standard and file format for specifying a simulation to be run over an SBML/CellML model. Can specify simulation algorithm and parameters.
libSBML and libSEDML	Import/export of respective standard–encoded files.
COMBINE	COmputational Modeling in BIology NEtwork (COMBINE) is an initiative to coordinate the development of standards (such as SBML, CellML, and SED–ML) and formats for modeling in biology.
COMBINE Archive / OMEX	A zip file–like container for standards covered by COMBINE. Also known by the moniker OMEX (Open Modeling and EXchange format).
Antimony / PhraSEDML	Human–readable representation of standards (Antimony for SBML/CellML and PhraSEDML for SED–ML). Provided as software libraries that can interconvert between human–readable form and standard encoding.
nteract	Notebook viewer featuring code and narrative.
libRoadRunner	Library / Python package for simulation of SBML ODE and stochastic models.
Jupyter	An umbrella project for technologies for authoring, rendering, and supporting literate coding notebooks. Provides a message protocol which can be used by any language to interface with Jupyter notebooks.
Plotly [35]	A set of software packages for rendering interactive plots using web technologies (HTML/Javascript). Used by Tellurium to provide high–quality plots in the notebook environment.
Tellurium	Integrated Python environment that includes or supports all of the above technologies and standards.

Integration is a major design goal of Tellurium. Tellurium brings together many different standards and technologies. These include not only standards such as SBML and SED–ML, but also has human–readable representations of these standards which have their own unique names (Antimony and PhraSEDML respectively). In this table, we summarize the principal standards, terms, and technology used in Tellurium.

Two very important technologies indicated in Table 1 are Antimony and PhraSEDML. These are human–readable shorthand representations which can be converted to/from SBML and SED–ML respectively (additionally, Antimony also supports converting to/from CellML models, but elements may not necessarily have a one–to-one correspondence due to the fact that Antimony was built specifically to represent SBML). These technologies form the basis of Tellurium’s *inline OMEX* representation of COMBINE archives, which allows converting an entire COMBINE archive, including all contained models and simulations, into a human–readable form. An inline OMEX cell is comprised of Antimony models enclosed in model…end blocks and PhraSEDML instructions in the global scope. Since Antimony models are delimited, each model…end block is automatically converted into an SBML model. All PhraSEDML instructions at global scope are then converted into a SED–ML file, which can contain multiple simulations and tasks. In the case where Tellurium is used to import a COMBINE archive containing multiple SED–ML files, Tellurium will add the headers %antimony and %phrasedml before each Antimony/PhraSEDML block. The headers are followed by a pathname locating the file inside the COMBINE archive. While it is not necessary to embed multiple SED–ML files in a COMBINE archive due to the fact that SED–ML can specify multiple simulations and tasks, the scheme outlined here allows Tellurium to gracefully interoperate in cases where multiple SED–ML files or a complex directory structure within the archive is desired.

**Fig 1:**
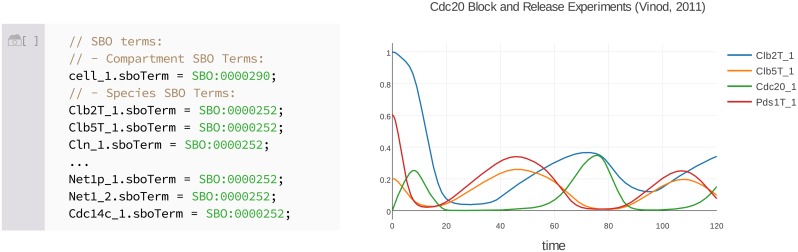
A demonstration of Tellurium’s SBO syntax. This figure shows a model of mitotic exit in budding yeast [38] available via the Biomodels repository entry BIOMD0000000370 [39]. When the SBML for this model is imported, Tellurium automatically extracts SBO identifiers for species, reactions, compartments, etc. and embeds the identifiers in the Antimony code. These identifiers point to specific physical, biological, or mathematical entities recorded in the ontology. For example, the first identifier, SBO:0000290, refers to a compartment in physical space [40]. Other identifiers refer to polypeptide chains (SBO:0000252) and protein complexes (SBO:0000297). These identifiers appear inline in the notebook cell and can be edited by the user. The right panel shows the transient response for this model from 0 to 120 minutes. This example is included with the Tellurium notebook viewer version 2.0.14 and later (*File→Open Example Notebook→Mitotic Exit (Vinod)*).

An often overlooked aspect of encoding models and simulations is connecting the mathematical entities represented by the processes in the model to physical, biological entities. This can be remedied by encoding semantic content in models using the Systems Biology Ontology (SBO) [36]. SBO is a vocabulary that contains a number of identifiers for linking mathematical entities in models to specific biological entities such as enzymes and substrates. SBO can also be used to describe mathematical concepts such as Michaelis–Menten kinetics [37]. Antimony was developed before the widespread use of SBO, and so lacks native support for SBO annotations. However, we have implemented support for SBO annotations as part of Tellurium’s inline OMEX syntax. Fig 1 shows an example of Tellurium’s SBO syntax. These SBO terms are preserved when the SBML or COMBINE archive is exported.

Tellurium’s design is defined by three major features: providing software libraries necessary for supporting reproducibility, integration of the steps in the workflow such that the steps can be performed automatically, and providing an interface for authoring models and visualizing the output of simulations. Table 1 shows the respective software libraries that are used to perform these tasks.

Tellurium’s notebook interface, based on the *nteract* app [41], allows authoring and editing of human–readable standards in a human–readable representation. The nteract environment is similar to Jupyter and supports Jupyter notebooks, but nteract is a complete redesign of the notebook viewer front–end using the Electron framework [42], which allows Web technologies to be used for desktop app development. Whereas Jupyter is a browser–based front–end, nteract is a desktop app, which carries several benefits for interaction with the host operating system [43]. Unlike Jupyter, nteract features: an installer and native file menus, 2) a terminal-free installation procedure, 3) integrated support for publishing notebooks to GitHub, and 4) additional user interface (UI) features, such as sticky cells and hidable cell input/output. nteract also aims for a more minimalistic user experience.

## Results

We demonstrate the benefits for reproducibility provided by Tellurium Notebook with case studies. In the first case study, we show how to encode multiple parameter sets and plot phase portraits. Explorations of parameter space are frequently done to determine if a model is applicable to conditions beyond those in the original model, an important consideration for testing model validity. The second case study evaluates if a model implementation produces results that are comparable to those in the original study via a series of tests which cover important dynamical properties of the model.

### Case study 1: Encoding post–processing and multiple parameter sets in combine archives

In order to meet our requirements for reproducibility, it is necessary to visualize different types of data, such as phase portraits. Furthermore, it is not sufficient to merely recreate a simulation. Rigorous reproducibility requires the ability to test existing models under a variety of circumstances (encoded as parameter sets). This first case study shows how Tellurium can be used to encode multiple parameter sets in a COMBINE archive. It also shows how COMBINE archives can be used to create phase portraits, which plot the transient value of one system variable against another.

For this case study, we use a model of M phase control [44]. M phase is triggered by the heterodimerization of cyclin (specifically the cyclinB in this model) and a cyclin–dependent kinase (Cdk) to form M phase promoting factor (MPF). MPF has an activating threonine phosphorylation site and two inhibitory phosphorylation sites (in this model, the two inhibitory sites are represented as a single inhibitory site) on the Cdk subunit. MPF is also regulated by the phosphatase Cdc25 (which activates MPF) and the kinase Wee1 (which inactivates MPF). MPF itself inhibits Wee1 and activaes Cdc25. Hence, it forms a pair of positive feedback loops with its own regulators. The model contains 21 reactions and is available via the BioModels repository (BIOMD0000000107 [45]).

**Fig 2:**
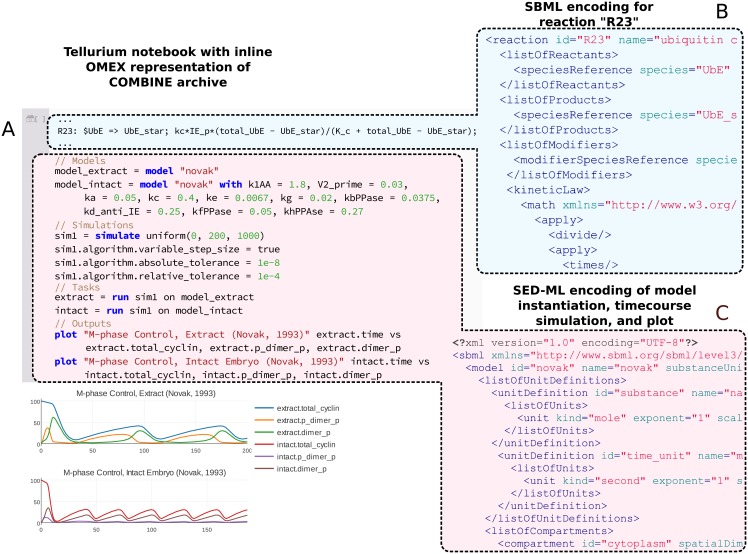
A comparison of Tellurium’s human–readable representation of a COMBINE archive shown in a Tellurium notebook (A) and excerpts from the equivalent SBML (B) and SED–ML (C) encodings. Tellurium’s in-line OMEX format contains human–readable representations of both SBML and SED–ML (A). Here, SBML is represented by Antimony code (with the definition of a single reaction in blue) and PhraSEDML (in red). (B) shows the SBML encoding for a single reaction. The single–line human–readable form of this reaction is highlighted in part (A) for comparison. The components of the Antimony syntax are as follows: R23 is the reaction label, the reactant $UbE, with a dollar sign indicating a boundary species, a => symbol, which indicates an irreversible reaction (reversible reactions can be indicated with ->), the product UbE_star, and the kinetic law comprised of everything following the semicolon. Using the SBML encoding, it is difficult to modify the reaction stoichiometry or kinetic law, whereas this task is easy in Tellurium. Finally, (C) shows the SED–ML encoding corresponding to the human–readable simulation portion of this COMBINE archive. The simulation portion performs the following functions: first, two SBML models are instantiated with different sets of parameters (in the original publicatuion [44], the authors provided one set of parameters for oocyte extract and a different set for intact embryos). Second, a timecourse simulation with an adaptive step size is attained by setting the variable_step_size property of the simulation as well as appropriate tolerance values. Finally, the simulation is run with the two different model instantiations and plotted with representative state variables (active MPF, doubly phosphorylated/inactive MPF, and total cyclin) to show the behavior of the two parameter sets.

Fig 2 shows a comparison of Tellurium’s human–readable representation of the M phase control model and simulation versus the standard–encoded representations. Clearly, readability is essential for model transparency. However, readability is essential for model reuse as well. To demonstrate this, we convert this SBML–only model into a COMBINE archive containing both SBML portions describing the model and SED–ML portions describing the simulation. We then show how Tellurium’s human–readable format permits easy modification of the published model and simulations contained in the COMBINE archive.

**Fig 3:**
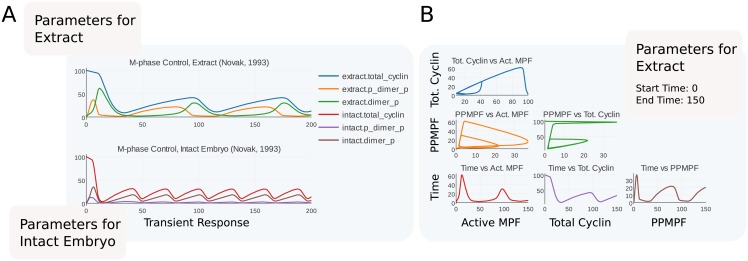
A demo of an SBML/SED–ML encoding contained in a COMBINE archive showing two useful features of the encoding: Multiple parameter sets (A) and post–processing (B). (A) Transient responses of M phase control [44]. This model was published with two parameter sets. One set is based on measurements from *Xenopus* oocyte extracts (top) whereas the other is based on measuremetns from intact embryos (bottom). (B) Phase portraits of representative state variables in the model. These variables are chosen after [44] and are as follows: total cyclin, doubly–phosphorylated MPF (PPMPF, the predominant inactive form of MPF [44]), active MPF, and time. Each pair of variables is plotted in this matrix. Y–axis variables are indicated in the rows of the plot and x–axis variables are indicated in the columns. The title of each subplot is given in terms of *x* vs *y*, e.g., the top left subplot shows total cyclin on the x–axis vs active MPF on the y–axis. Phase portraits can show transients (such as the initial response of total cyclin in the upper left corner in blue, which starts at 100 and decreases to its normal range) as well as limit cycles (exhibited by all three phase portraits in the upper part of (B)). The slope of a given region of the phase portrait is useful for showing the relative rate of change of two quantities. The green and orange curves show regions where one quantity changes rapidly with respect to another. These regions correspond to the rapid rise in active MPF due to positive feedback from MPF to its own self–activation, and the subsequent falloff of total cyclin due to cyclin degradation via a ubiquitin pathway activated by MPF. The plot in part (B) is derived from the parameter set for oocyte extract, corresponding to the top plot in part (A).

In order to create a SED–ML specification for this model, we need to define four steps in the workflow, which correspond to distinct elements in SED–ML: (1) model definition, (2) simulation, (3) task specification, and (4) output generation. For (1), models can be defined in Tellurium’s human–readable format by referencing SBML or CellML files in the same COMBINE archive, with the option of including parameter replacements. For (2), SED–ML simulations can be either timecourse simulations or steady state computation, and can reference a specific algorithm (e.g., LSODA), or a generic implementation using KiSAO [20]. Tellurium uses predefined keywords such as lsoda (an ODE solver implementation [46]) to refer to popular implementations. In SED–ML, simulations are specified independently from models. This allows model and simulation elements to be reused in different combinations. For (3), SED–ML uses task elements to describe these combinations. Finally, the output elements of (4) can be plots or reports and allow users to access the output of tasks. Tellurium’s human–readable format allows defining a SED–ML model by instantiating the same SBML model with different parameter values (*m* in this example) using the syntax:

mymodel = model “novak” with **param1 = value2, param2 = value2** …

with the param/value pairs being replaced by the corresponding parameter ids and values respectively. We use this syntax to instantiate two copies of the model, one with parameter values for extract and another with parameter values for intact embryos [44]. Finally, we show how Tellurium can be used to encode integrator tolerances and encode an adaptive step–size simulation in SED–ML. Fig 3 shows simulation results for both parameter sets.

This case study shows that Tellurium provides an efficient means of converting SBML models into exchangeable COMBINE archives containing simulation components. Furthermore, COMBINE archives can contain important dynamical information about the model, such as the behavior under the different parameter sets that we explored in this study.

### Case study 2: Reproducibility through in–depth variational studies

Reproducibility requires that a model implementation produces results consistent with the original study, especially if a different authoring tool is used. In order to provide criteria for judging whether a model reproduction is consistent with the original, a set of testing criteria is required, similar to the concept of unit testing in software. However, researchers seldomly perform extensive checks on the dynamics of models before using them. This is due in part to the lack of tool support for easily modifying and producing variants of models and simulations encoded in exchangeable formats. Tellurium’s authoring features enable modelers to encode dynamical unit tests in COMBINE archives, thereby providing a way to verify that a model has been correctly reproduced.

For this case study, we reproduce a highly–detailed model of syncytial nuclear divisions in the *Drosophila* embryo [47] through testing the model’s dynamics under different conditions. This model is biologically similar to the model in Case Study 1 [44] but is more detailed (54 vs. 23 reactions) and is available as a pre–encoded COMBINE archive [48]. In many insect species, the embryo enters a period of rapid mitotic division without cytokinesis [49] immediately following fertilization. In *Drosophila*, 13 of these divisions occur within 3 hours of fertilization [47]. MPF is again the main regulator of these divisions. Recalling that MPF is a heterodimer of cyclin and Cdk, cyclin subunits tend to be the limiting factor in complex formation, and are thought to regulate mitotic division. Cyclin availability is controlled by the anaphase promoting complex (APC), which targets CycB for degradation. However, in *Drosophila*, the levels of CycB appear to remain high during the first 8 mitotic divisions [50]. This observation can be reconciled with known mechanisms by assuming that CycB degradation only occurs in the vicinity of the mitotic spindle [47, 51, 52], despite the absence of a nuclear envelope during the mitotic divisions. To account for this hypothetical local degradation of CycB, the model artificially separates the cytoplasm into two “compartments,” with a cytoplasmic compartment representing the cell and a nuclear compartment representing the volume in the vicinity of the mitotic spindle.

**Fig 4:**
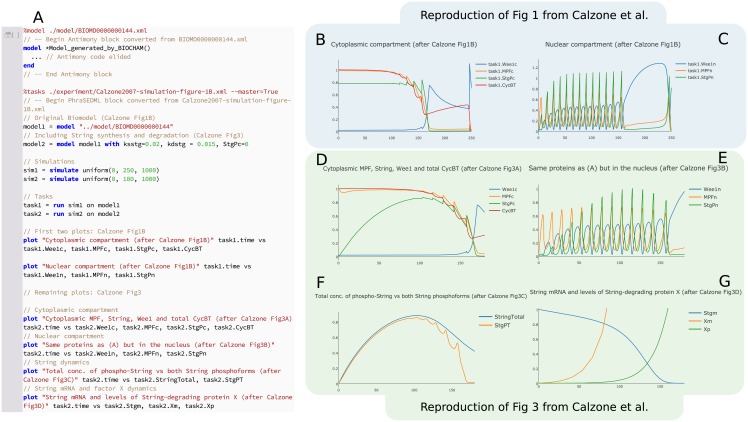
Using Tellurium to reproduce model variants in [47] and repackage as a COMBINE archive. To demonstrate the use of COMBINE archives for encoding model variants, we began with a COMBINE archive describing a single variant of this model without String synthesis or degradation [48], which reproduces Fig 1B of [47] (plots B and C here). We then used Tellurium to add a variant describing String degradation, which reproduces Fig 3 of [47] (plots D through G here). Panel (A) shows the inline OMEX cell with the Antimony code elided (it would belong in the model Model_generated_by_BIOCHAM…end block, where the ellipsis is currently shown). Everything after the end instruction is thus PhraSEDML. Plots B and D show the transient response of the cytoplasmic compartment of the model. Plots C and E show the nuclear compartment (defined as the spatial region around the mitotic spindle). Plot F shows the levels of total String and its phosphorylated state. Plot G shows the level of String mRNA and protein factor X, which degrades String mRNA. Note the y–axis scale on plot G was manually adjusted to show the mRNA dynamics. The subplots in this figure intentionally have different durations, after Calzone et al [47]. The model in [47] was authored using BIOCHAM [53]. Our model reproductions that reproduce these plots are available as a COMBINE archive [54].

As a starting point, we use the COMBINE archive encoding of this model by Scharm and Tourè [48]. This archive contains SBML derived from biomodel BIOMD0000000144^1^, which is intended to reproduce Fig 1 of [47]. However, the archive does not contain more extensive tests of the model’s dynamics, such as whether the model can be used to reproduce several other simulations described in the paper. The initial variant encoded by the COMBINE archive and shown in Fig 4B and 4C is based on a model with a constant level of the phosphatase String (which corresponds to a Cdc25 homolog in Drosophila), whereas in reality String levels change over the course of the mitotic cycles. String regulates MPF via a positive feedback loop, and has been shown to peak at the seventh or eighth cycle of the mitotic divisions [47]. To account for this, Calzone et al. [47] posited that String mRNA is degraded by a hypothetical factor “X,” causing the synthesis rate of String to drop over time. Therefore, we have modified the SED–ML of the original COMBINE archive [48] as follows to include the synthesis and degradation of String. We are able to reproduce Fig 3 of [47] by making these modifications to the original COMBINE archive:

- Enable synthesis and degradation of String by setting the parameters ksstg = 0.02 and kdstg = 0.015 respectively.
- Set the initial concentration of total String to zero by setting StgPc = 0.
- Compute the total amount of unphosphorylated String by adding the rule
StgT ≔ (1 − N*E_1)*Stgc + N*E_1*Stgn.
- Compute the total amount of String in the cell by adding the rule
StringTotal ≔ StgPT + StgT.

**Fig 5:**
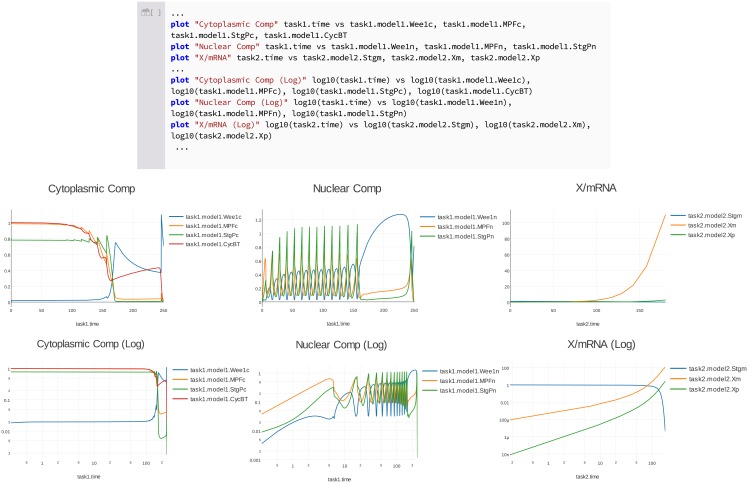
Comparison of logarithmic (bottom row of plots) vs. linear (top row of plots) plotting of the transient response in Fig 4B, 4C and 4G, along with an excerpt of the relevant PhraSEDML code for plotting on logarithmic axes. Fig 4G, which is a plot of String mRNA and hypothetical factor X, which degrades String mRNA, exhibits a large dynamic range. Logarithmic plotting helps visualize the dynamic range of these quantities. This is achieved in PhraSEDML by wrapping the quantities for x and y axes inside a log10 operation.

Tellurium makes it easy to encode both the original variant, without String synthesis and degradation, and the variant including these terms in a COMBINE archive [54]. Fig 4 shows the results of executing this COMBINE archive in Tellurium, and Fig 5 shows logarithmic plots of the transient response. We have thus expanded the dynamical test cases for this model, as it now reproduces two simulations from two different variants described by the original authors (Fig 1 and 3 of [47]), enabling better coverage of the model’s dynamics.

In order to gain insight into the regulatory mechanism controlling the mitotic divisions, and understand the transitions that control the exact number of these divisions, Calzone et al. performed a one–parameter bifurcation analysis [47]. Bifurcation analysis probes the number and position of steady states and other types of attractors as a function of a parameter. The oscillations shown in Fig 4 are the result of discrete division events, and the behavior shown does not represent a limit cycle. However, the model can be shown to exhibit limit cycle behavior by 1) removing all discrete events and 2) fixing the number of divisions by introducing the variable *C* as a cycle counter. The number of nuclear compartments is then given by *N* = 1.95^*C*^ (1.95 is a scaling factor described in [47]). For a given cycle number *C*, MPF exhibits limit cycle oscillations, although the amplitude and period of these oscillations changes with the cycle number. At low cycle number, Calzone et al. observed that these oscillations were dominated by the negative feedback effect of cyclin degradation, whereas for large cycle number (*C* ≥ 12), positive feedback via control of phosphorylated MPF by the kinase Wee1 and phosphatase String contributes to the oscillations.

**Fig 6:**
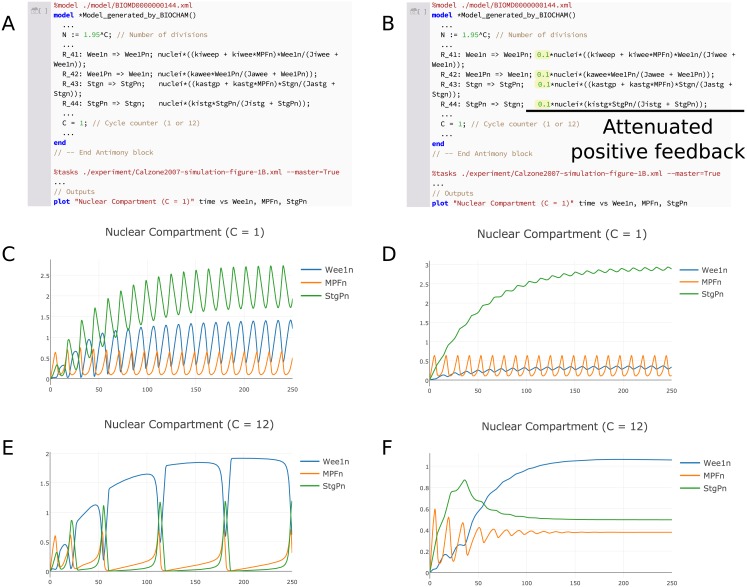
Testing the shift in regulatory mechanism of mitotic oscillations. To verify the observation [47] that the number of mitotic divisions in the *Drosophila* embryo is governed by a shift from negative to positive feedback, we first removed all discrete events and introduced the variable *C* such that *N* = 1.95^*C*^. We then compared the limit cycles produced by this eventless model (left) with those produced by a variant with attenuated positive feedback from the regulators Wee1 and String (right). Attenuation was achieved by decreasing the rates of the phosphorylation and dephosphorylation of Wee1 and String. The original model exhibits stable limit cycle oscillations for both early cycles (C), which are putatively dominated by negative feedback, and late cycles (E), which are putatively dominated by positive feedback. The attenuated model only exhibits stable oscillations at early cycles (D), suggesting that positive feedback does indeed play a role in late cycle oscillations (F). Our model reuse and modification study is available as a COMBINE archive that reproduces the figure shown and facilitates further modification and reuse [55].

SED–ML does not support bifurcation analysis, precluding us from reproducing that part of the study in an exchangeable format. However, it is still possible to test the change in regulatory shift from negative to positive feedback. Instead of a bifurcation diagram, we compare the limit cycle behavior of the original model to a model variant with reduced Wee1 and String activation and deactivation rates. This slows the timescale of the positive feedback component of the model. Fig 6 compares the behavior of the original model at early and late cycle numbers with the variant containing attenuated positive feedback. Whereas the normal model exhibits stable limit cycle oscillations at both *C* = 1 and *C* = 12, the oscillations in the attenuated model are transient at late cycles (*C* = 12) but not at early cycles (*C* = 1). This observation suggests that String and Wee1 dynamics are indeed crucially important for late cycle oscillations, but not for early cycle oscillations, confirming the shift in regulatory mechanism. These simulations thus form a third set of unit tests for the model, encoded as a COMBINE archive [55].

In summary, using Tellurium’s editing capabilities, we have created an extensive set of unit tests for dynamical behavior of this model, which we exported as a COMBINE archive and imported into another tool as shown in S1 Fig. Creating these tests required a means of quickly editing and expanding upon both the SBML and SED–ML embedded in the COMBINE archive. Tellurium’s notebook approach allows us to satisfy these requirements, and provides an integrated workflow for testing the dynamical behavior of the model.

### Interoperability concerns & test cases

In order to achieve the exchangeability requirement of reproducibility, broad standards compliance is necessary. A small number of test cases, such as the first two case studies, is not sufficient to ensure interoperability with other software. During Tellurium’s development, we gathered a number of COMBINE archive exemplars from literature, other software tools, and our own archives. We have provided these archives as a resource to other developers by making them publicly available online. The test archives are structured to separate examples with advanced SED–ML features from those with basic SED–ML usage, enabling tool developers to implement incremental support for the standard. S1 Table lists all test archives and how to obtain them.

### Advanced SED–ML support

In order to address the requirement of broad standards compliance, we tested Tellurium against a set of tests provided by the SED–ML Web Tools [27]. These tests utilize advanced features of the SED–ML standard, and are designed to demonstrate the standard’s coverage of different types of analysis. S2 Table lists all files used in this test set, and S4 Fig shows the results of exporting these files to Tellurium and back again.

### SBML test suite COMBINE archives

The SBML Test Suite [56] is a collection of dynamical models along with expected trajectories designed to test software tools for compliance with the SBML standard. Each test case contains a SBML model, simulation parameters encoded in SED–ML, expected trajectories encoded as a comma–separated values (CSV) file, and graphical plots for reference. We converted each of these 1196 test cases into COMBINE archives containing the SBML models, SED–ML simulations, and CSV expected results and used these COMBINE archives as a benchmark for Tellurium’s support for standards. The results of this benchmark are shown in S3 Table.

## Discussion

In order for the conclusions of a research study to be valid, the models used in the study must be reliable. Using SED–ML to reproduce the dynamics of a model and compare these dynamics with expected values adds crucial value to the integrity and validity of studies that reuse or expand on the model. As an exchangeable format, SED–ML is confined to the intersection of the most common features available in dynamical modeling tools, which leaves out certain useful types of analysis (e.g., bifurcation analysis). However, we argue that the use case of SED–ML is not to serve as a replacement for current analysis methods. Instead, SED–ML is a tool to test the dynamical behavior of models before using them. For example, while we were not able to reproduce the bifurcation analysis of the mitotic division study [47] in an exchangeable format, we were able to verify the observations regarding the shift in regulatory mechanism, and in doing so gained new insight from this alternative approach. A researcher may also wish to verify that the model reproduces certain expected behaviors. For example, if the model is expected to exhibit switch–like behavior, does this behavior occur at the correct input threshold? For models with feedback, such as integral feedback control [57], does the output exhibit robustness in the presence of perturbations? These types of validation require expert knowledge of the system. While there are tools and resources to help with this, the most important point for conveying this information to other researchers is to encode it as transparently and lucidly as possible, which is achieved using the literate notebook approach described here.

Tellurium’s approach of blending standards with literate coding enables researchers to create rich, detailed workflows incorporating community standards. Tellurium allows the models and simulations from these notebooks to be shared with other tools via COMBINE archives. This allows other users to import these models and simulations and reproduce them using independently developed software tools. This is consistent with our original definition of reproducibility, as it enables robust cross–validation of results between tools, as opposed to simply repeating a previous simulation. It also helps ensure that the tools themselves are accurate and free of idiosyncrasies that could affect the analysis results. Model repositories such as BioModels [58, 59], JWS Online [60, 61], and the CellML model repository [62] have enabled widespread support for the SBML and CellML standards. We believe that better tool support for SED–ML and COMBINE archives will help create a trend toward better adoption of these formats by repositories.

### Comparison with existing software

Many dynamical modeling tools support exchanging models via the SBML format, including COPASI [63, 64], SBW [65], iBioSim [66], PathwayDesigner [67], CellDesigner [68, 69], VCell [70–72], CompuCell3D [73], PySCeS [74], BioNetGen [75], and PySB [76]. These tools have diverse feature sets and intended use cases, such as tissue modeling (CompuCell3D), rule–based modeling of molecular complexes (BioNetGen, PySB, VCell), and general modeling and simulation (all others). The tools also have different forms of user interaction, such as graphical user interfaces (COPASI, iBioSim, VCell) and graph–based network editors (CellDesigner, PathwayDesigner). Python–based tools such as PySCeS [74] and PySB [76] can be used with a Jupyter notebook, but do not feature integration of standards with the notebook itself. In general, Tellurium is useful when the user wishes to interactively edit and test standard–encoded models and simulations or produce presentations and PDFs of modeling studies.

Tellurium’s Python foundation makes it easy to combine with other Python–based software such as PySCeS, COBRApy [77], and PySB. There are also many specialized Python packages for specific tasks such as moment closure approximation for stochastic models [78], parameter estimation [79], Bayesian inference [80], and estimating rate laws and their parameter values [81].

In biomedical research, certain tools have been created specifically to facilitate reproducible research. One such tool is Galaxy [82]. Galaxy is a web–based tool which allows users to create workflows describing experiments, e.g., metagenomic studies [83]. A similar tool with a focus on web services and which supports SBML–based workflows is Taverna [84]. Galaxy and Taverna allow users to annotate each step of the workflow, which provides a way for others to follow and understand the chain of reasoning used in the workflow’s construction. This satisfies the requirement of transparency, as it allows users to view the sequence of steps used to produce a result. Although this approach is very different from a literate notebook in terms of the way the user interacts with the system, it shares the goal of allowing the user to see the sequence of steps used to produce a result and interrogate the specific procedure used in each of the steps. Galaxy and Taverna also allow users to share workflows via the web. However, neither tool attempts to directly address the problem of exchangeability with other software tools.

VisTrails [85] is another workflow system based on visual design. VisTrails focuses primarily on generating rich, three–dimensional diagrams and visualizations based on input data and a specific sequence of steps. VisTrails also saves all changes made to a workflow and allows users to view previous versions, a concept termed “retrospective provenance” [86]. However, this approach also lacks exchangeability. Furthermore, while graphical tools may be more accessible because they abstract away the underlying algorithms, it can be difficult to isolate and correct software errors when a step fails due to bad input or an internal error.

Many other research software systems make use of notebooks, and some incorporate special extensions. StochSS [87], the GenePattern Notebook [88], the SAGE math system [89], and the commercial Mathematica software [30] all utilize notebooks which are specially tailored or feature special extensions for each respective application. However, none of these approaches attempt to solve the problem we address: workflow integration with exchangeable standards. Our usage of the literate notebook approach is intended to satisfy two specific requirements, which are distinct from other use cases: 1) to make these standards easy for humans to read, understand, and modify, without requiring expert knowledge of the technical specifications of the standards, and 2) provide an integrated workflow which facilitates exchangeability with other software.

The notebook approach used by Tellurium also has disadvantages. For example, while notebook files can be stored in a version control system, diffing and merging these files is difficult because the files are not line–based. Furthermore, large or complex analyses can be difficult to orchestrate using notebooks, as interacting with a large notebook with many cells can be cumbersome. For this reason, we also distribute Tellurium as a set of Python packages. When a workflow becomes too difficult to manage in a notebook environment, users can easily resort to separate Python, Antimony, and inline OMEX files in order to include the existing work in a more manageable system or to include the work in a version–controlled repository. Nevertheless, we believe that Tellurium’s approach is highly useful in many crucial use cases, including testing models, experimenting with model variants, and as a final step in producing an analysis for other researchers in a transparent, visual presentation.

## Conclusion

In order to build larger, more complete, and more accurate dynamical models of cells and tissues, it will be necessary to reuse models of subsystems. This is currently very difficult due to the time–consuming and laborious process of manually reconstructing models from literature, or manually verifying third–party SBML models. Tellurium provides support for encapsulating both a model and its dynamics in a community–developed standard format, the COMBINE archive. This archive can contain the model as well as a number of simulations which test various dynamical properties of the model. Tellurium allows users to create COMBINE archives easily from SBML models, or import and modify preexisting COMBINE archives.

Tellurium integrates SBML, SED–ML, and COMBINE archives within a notebook environment, making it exceptionally easy for users to work with these standards, and obviating the need for users to understand the technical specifications of the standards. The availability of authoring tools such as Tellurium will make it possible for model repositories to begin implementing support for SED–ML and COMBINE archives. Indeed, the JWS Online repository [60, 61] already has support for exporting COMBINE archives of models and simulations, which can be read by Tellurium. We hope that other databases will follow suit so that it will be possible to automatically extract dynamical information from these repositories.

Tellurium’s human–readable representation of COMBINE archives is highly important for facilitating model modification as we describe here. This feature enables researchers to experiment with models using alternate parameterizations in order to test the dynamical behavior of the models under varying conditions. We hope that this will lead to more robust models which lead to biological insight by providing predictions under a wide range of circumstances, as with the case studies presented here.

## Availability and future directions

Tellurium Notebook is available as a standalone app (tellurium.analogmachine.org) or as a collection of Python packages hosted on the Python Package Index (pypi.python.org) for 64-bit versions of macOS, Windows, and Linux. The Tellurium Python packages support Python 2.7, 3.4, 3.5, and 3.6. The notebook app comes bundled with Python 3.6 and all requisite packages. The source code of Tellurium (github.com/sys-bio/tellurium) is licensed under the Apache license, version 2.0. Tellurium incorporates or makes use of other software, such as *nteract*, *Plotly* (http://plot.ly), *Python*, *libSBML*, *libSEDML*, and others, which are licensed under their respective terms. See tellurium.analogmachine.org for links to installation instructions, documentation (tellurium.readthedocs.io), and the source code (github.com/sys-bio/tellurium).

There is a clear need to support exchangeability of simulation experiments in order to allow researchers to build larger, better tested, and more comprehensive models. Tellurium’s built–in support for exchangeability comes from the SBML and SED–ML standards. This allows Tellurium to support the widest possible range of software tools, but also prevents exchanging studies not covered by SED–ML’s vocabulary of predefined simulation types. Due to delays associated with standardizing and implementing features, SED–ML tends to lag several years behind other systems which do not rely on standardization. Thus, SED–ML has the advantage of stable support from a wide range of tools, but has the disadvantage of lacking the flexibility to encode custom studies based on recent advancements in model simulation.

In order to provide a more flexible platform for encoding simulation studies, new solutions are needed. One such solution would be to extend SED–ML with generic scripting capabilities. Another solution would be to build an alternative platform for exchanging simulation experiments. For example, the SESSL [90] software tool also provides a means for encoding and exchanging simulations. Whereas SED–ML uses a standardized XML schema to describe simulations, SESSL uses a domain–specific–language implemented using the Scala programming language. This allows users to mix in Scala code to access features not yet available via SESSL’s public interface. However, this approach is not language–agnostic and is tied to Scala and its low–level execution engine. The SED–ML standard, in contrast, does not constrain the low–level operation of its implementations.

In this paper, we have argued for modelers to construct “unit tests” for dynamical models by including model variants as in the study by Calzone et al. [47]. We have shown that these variants are easy to construct and encode in COMBINE archives using Tellurium, but we have not addressed how to validate these tests in an automated way. Due to simulation algorithm differences between tools and the presence of multiple steady states in some models, performing a direct numeric comparison between steady state values or timecourse traces may be too fragile to be useful.

The BIOCHAM software tool [53] employs an interesting solution by using temporal logic constructs to make assertions about properties of model timecourse dynamics. Using this approach, it would be possible, for instance, to make semi-quantitative assertions such as “species X exhibits oscillations with a period of 100 ± 50 mHz”. These logical constructs could be used in lieu of a direct numerical comparison to validate the dynamics of a model. A practical solution to the problem of validating model timecourse dynamics would likely make use of semi-quantitative assertions such as “Is the number of oscillations of X at least 10,” “Does Y exhibit a peak value of at least 100 nM,” or “Does the response time of the system fall within a certain range?”

However, we believe that several important questions remain before such a validation method will be useful in practical contexts, such as what is the minimal set of formal logic expressions sufficient to capture any useful assertions, and what are the best practices for encoding assertions? For example, should the assertions strive to use relative relationships between model quantities, such that reparameterizing the model does not affect the assertions, or should they be valid only for a single given parameterization? In the former case, how should model variations be generated to test assertions? We believe that implementing automated testing of dynamical models requires addressing these questions in a well thought–out way. Until then, we believe that manually comparing results encoded as COMBINE archives as in the studies presented here will provide immediate benefits to reproducibility. For moderate–size models such as the Calzone study, we have shown that this approach is a practical solution.

In addition to reproducibility, other important problems remain to be tackled. One often overlooked application area of software tools and methods is teaching. The teaching burden for the next generation of systems biologists is greater than it has ever been. As members of an integrative field, systems biologists must master a variety of mathematical and computational techniques, possess intimate familiarity with many biological systems, and must increasingly be familiar with standards and specifications. Part of this burden can be eased by embracing new software technologies for interactive teaching. Tellurium’s notebook format makes it suitable for creating interactive exercises and lessons. The ability to edit code snippets, replot results, and use the built–in markdown and L^A^T_E_X rendering features of Tellurium/nteract makes notebooks a powerful teaching tool. Moreover, Tellurium’s connection to standards helps ease the learning burden for researchers who wish to encode their models and simulations in standard formats, thereby helping to ensure future reusability of this important community resource.

While Tellurium’s approach of blending Antimony and PhraSEDML has the benefits of allowing users to author and edit COMBINE archives without becoming experts in standards, it does have drawbacks. One such drawback is that mixing two different languages can create ambiguities if either language gains new syntax. A better solution could be to design a single, unified language targeting multiple standards. This would be a greater technical challenge than designing a human–readable representation for a single standard and would not necessarily exhibit a one–to–one mapping between its syntax and the underlying standards, but it would allow for evolution of the syntax and also provide better integration.

Increasingly, systems biologists must rely on many different software technologies. We have found that a major bottleneck to productivity in dynamical modeling is integrating these technologies into a pipeline that can import and export standards, communicate with other scientific computing packages such as Numpy and Pandas [91], and make use of cutting–edge technologies such as nteract and Plotly for user interaction. Indeed, Tellurium represents much of our effort over the past few years of bringing these technologies together to serve as a useful platform for modeling. However, one aspect of integration we have not yet addressed is the ability to interoperate with other Python packages for simulation and modeling. One of our future goals is to support other Python–based SBML simulators. Due to the many packages in SBML, it has become unusual for a simulation to support all types of models that can be encoded in SBML. Therefore, in order to be able to support all types of models, it will be necessary to make use of different simulators. There is currently no common format for the output of these simulators, so they cannot be easily integrated in the same simulation study. Providing a common point of convergence is one of our future goals for Tellurium.

## Supporting information

S1 Fig Demonstrating exchangeability of the second case study. To show that the extended set of simulations from Fig 4 can be exchanged with other tools via a COMBINE archive, we exported the study in Fig 4 to the SED–ML Web Tools and verified that the plots were identical to Fig 4.
(EPS)

S2 Fig Examples of Tellurium’s inline OMEX format for specifying COMBINE archives. These examples start with very simple cases and build on these cases with progressively more advanced features. The first example contains a simple two–species model and simulated using a deterministic and stochastic solver. The second example shows a phase portrait. The third example shows multiple stochastic traces. The fourth example shows a one–dimensional parameter scan. All of these examples are available via a Tellurium notebook, which can be accessed by clicking on “File” → “Open Example Notebook” → “COMBINE Archive Basics” from within the Tellurium notebook viewer.
(EPS)

S3 Fig A normative example of a COMBINE archive introduced in the original paper describing the COMBINE archive format [26]. This example contains the repressilator model [92], a damped oscillation variant showing the modification of model parameters using SED–ML, and a phase portrait of the undamped system.
(EPS)

S4 Fig Round-tripping the SED–ML Web Tools examples [27]. In order to demonstrate broad support for standards, we conducted a series of tests utilizing advanced usage of SED–ML. The first row shows the original example rendered in the SED–ML Web Tools. The second row shows the same example imported into Tellurium. The third row shows the simulation after editing the model in Tellurium. Finally, the fourth row shows the result of re–exporting the example to the SED–ML Web Tools using a COMBINE archive. These COMBINE archives are available from the SED–ML Web Tools [27] and from our repository [93].
(TIF)

S1 Table Combine archive test cases. We collected all COMBINE archives used during development of Tellurium in an online repository on GitHub [93]. These archives serve to test Tellurium’s standards compliance, but they may also allow other tool developers to better support COMBINE archives. We have therefore organized the test cases into different categories, from toy examples using progressively more advanced features of SED–ML, to BioModels, and finally advanced SED–ML usage. The test suite draws archives from a wide range of sources: publications [26, 94], other tools (e.g., the SED–ML Web Tools [27]), the SBML Test Suite encoded as COMBINE archives, and archives developed by our group. The COMBINE test suite contains archives ranging from basic examples to advanced usage of the SED–ML standard. To verify exchangeability, we have manually tested importing these archives into our software and also into the SED–ML Web Tools. The SBML test cases were too numerous to test in this way, so a subset of archives were tested with the SED–ML Web Tools whereas the full set of archives was tested with Tellurium using a Tellurium notebook [95].
(EPS)

S2 Table Advanced SED–ML tests (provided by the SED–ML Web Tools [27]). The SBML test suite was converted into COMBINE archives using the provided notebook https://github.com/0u812/tellurium-combine-archive-test-cases/blob/master/sbml-test-suite/convert-to-combine-arch.ipynb. These SBML test cases are automatically converted into COMBINE archives containing the expected results, which are then converted by Tellurium into inline OMEX and simulated. A “failed” test refers to a case where the numeric simulation results diverge from the expected values. An “unsupported” test refers to a test that uses features not available in our simulator (libRoadRunner) or the inline OMEX strings.
(EPS)

S3 Table Tellurium SBML test suite results. This table shows the results of converting the SBML test suite into COMBINE archives and running the test cases with Tellurium. Notebooks for running these tests and converting the SBML test suite to COMBINE archives can be found in our repository [96]. The SBML test suite was converted into COMBINE archives using the provided notebook convert-to-combine-arch.ipynb [97]. The tests were then run with the notebook run-tests.ipynb [95]. This notebook plots each test result, and hence should ideally be used for runs of 100 test cases or less, as in the above table. Another notebook run-batch.ipynb does not plot each result, and is better suited for running all tests [98]. This notebook is also used to run the performance benchmark. Performance results are reported as mean ± standard deviation using three runs each. The SBML test cases are automatically converted into COMBINE archives containing the expected results, which are then converted by Tellurium into inline OMEX and simulated. A “failed” test refers to a case where the numeric simulation results diverge from the expected values (a maximum MSE ≥10^−3^). An “unsupported” test refers to a test that uses features not available in our simulator (libRoadRunner) or the inline OMEX strings. Since libRoadRunner does not support algebraic rules, delays, or fast reactions in SBML, test cases for these features are unsupported. All tests were performed on a Dell Optiplex with an Intel i7-3770 CPU at 3.40 GHz with 8 GB RAM.
(EPS)

## Footnotes

^1^http://identifiers.org/biomodels.db/BIOMD0000000144
