# Supplementary figures and images for: Tellurium notebooks—An environment for reproducible dynamical modeling in systems biology
Source: PLoS Comput Biol. 2018 Jun 15;14(6):e1006220. doi: 10.1371/journal.pcbi.1006220 (PMC6021116; doi:10.1371/journal.pcbi.1006220)

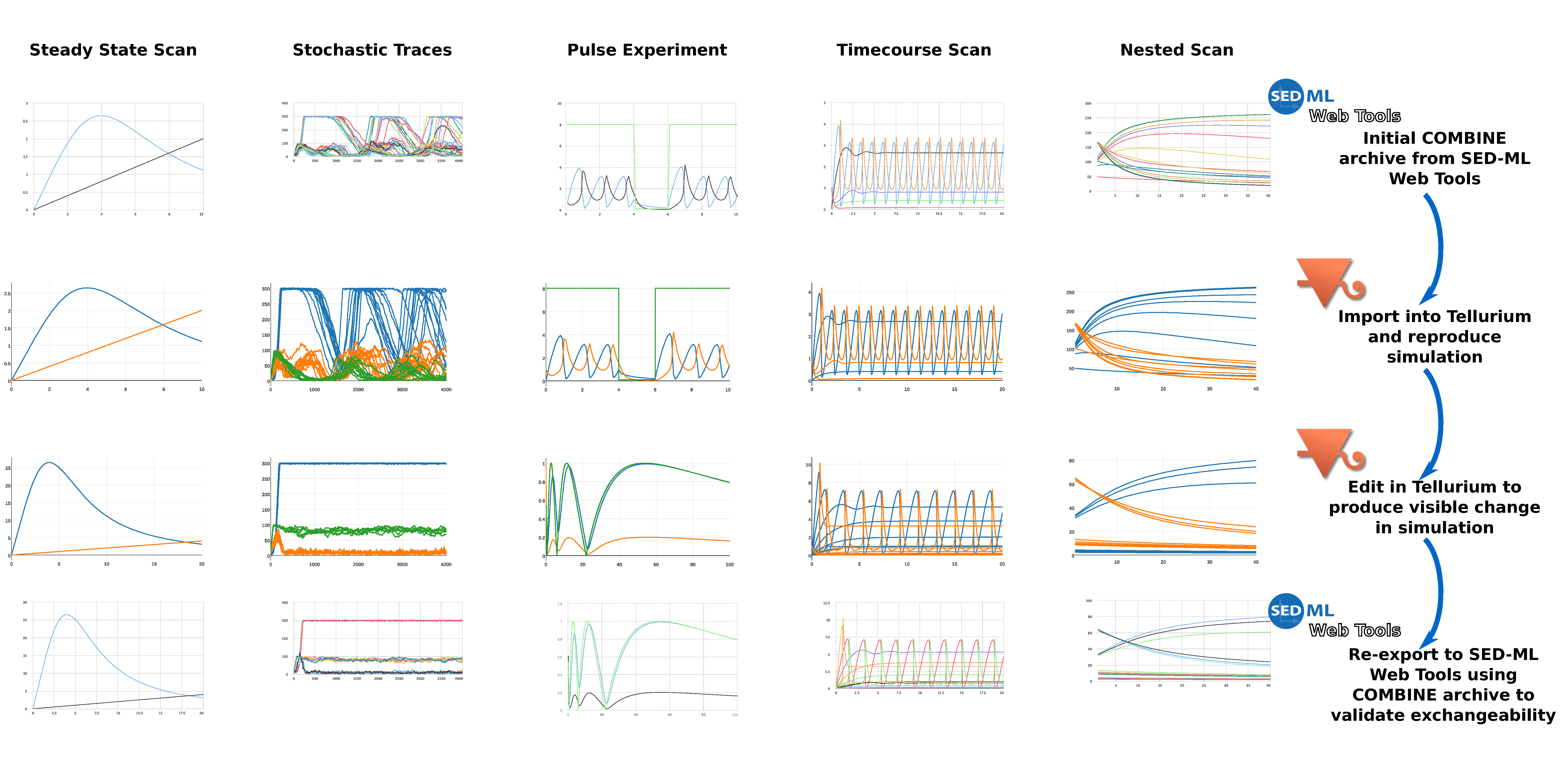

Supplement: S4 Fig — In order to demonstrate broad support for standards, we conducted a series of tests utilizing advanced usage of SED–ML. The first row shows the original example rendered in the SED–ML Web Tools. The second row shows the same example imported into Tellurium. The third row shows the simulation after editing the model in Tellurium. Finally, the fourth row shows the result of re–exporting the example to the SED–ML Web Tools using a COMBINE archive. These COMBINE archives are available from the SED–ML Web Tools [27] and from our repository [93]. (TIF) [file pcbi.1006220.s004.tif]
